# Cost-effectiveness analysis of XELOX versus XELOX plus bevacizumab for metastatic colorectal cancer in a public hospital school

**DOI:** 10.1186/s12885-017-3679-5

**Published:** 2017-10-17

**Authors:** Andrea Queiróz Ungari, Leonardo Régis Leira Pereira, Altacílio Aparecido Nunes, Fernanda Maris Peria

**Affiliations:** 1 Division of Pharmaceutical Assistance, General Hospital of Ribeirão Preto Medical School, University of São Paulo, Campus Universitário, s/n - Vila Monte Alegre, Ribeirão Preto, SP 14049-900 Brazil; 2 Department of Pharmaceutical Sciences, School of Pharmaceutical Sciences, University of São Paulo, Ribeirão Preto, Brazil; 3 Department of Social Medicine, Ribeirão Preto Medical School, University of São Paulo, São Paulo, Brazil; 4 Clinical Oncology Division - Internal Medicine, Ribeirão Preto Medical School, University of São Paulo, São Paulo, Brazil

**Keywords:** Colorectal Neoplasms, Cost-effectiveness evaluation, Bevacizumab, Unified health system, Brazil

## Abstract

**Background:**

Metastatic colorectal cancer imposes a substantial burden on patients and society. Over the last years, progresses in the treatment have been made especially due to the introduction of monoclonal antibodies, such as bevacizumab which, on the other hand, has considerably increased the costs of treatment. We performed a cost-effectiveness analysis of bevacizumab plus XELOX in comparison with XELOX alone in metastatic colorectal cancer in first-line therapy, from the perspective of a public hospital school in Brazil.

**Methods:**

This was a cost-effectiveness analysis performed by a decision tree and Markov models. Costs were expressed in local currency and outcomes were expressed in months of life gained. The model was constructed using the TreeAge Pro 2013® software.

**Results:**

The incremental difference in years of life gained was 2.25 months, with an extra cost of 47,833.57 BRL, resulting in an incremental cost-effectiveness of 21,231.43 BRL per month of life gained.

**Conclusions:**

Although the XELOX plus bevacizumab regimen is a more expensive and more effective treatment than XELOX, it does not fit the reimbursement values fixed by the public healthcare system in Brazil.

## Background

Colorectal cancer (CRC) is the third most common type of cancer among men and women in the world. In 2016, an estimated 95,270 new cases of colon cancer and 39,220 new cases of rectal cancer are expected to be diagnosed, and 49,190 people will die from CRC [1]. Although CRC is diagnosed at early stages in most cases, leading the possibility of curative surgical procedure, nearly 20% of patients suffer from metastatic disease at the moment of diagnosis [2] and, in this case, treatment is not considered curative.

Fluoropyrimidine 5-fluorouracil (5-FU) was the first drug to emerge as the drug of choice for metastatic colorectal cancer (mCRC). Later, in the 90 decade, two additional agents – irinotecan and oxaliplatin showed anticancer activity in mCRC [3].

Angiogenesis is a vital process for the progression of primary tumors and metastasis, and new therapeutic approaches for mCRC have focused on the inhibition of this process. Bevacizumab is a humanized recombinant monoclonal antibody which blocks the activity of all isoforms of vascular endothelial growth factor (VEGF), one of the main proangiogenic growth factors. The neutralization of VEGF biological activity reduces tumor vascularization and inhibits tumor growth [4].

Meta-analyses of randomized, clinical trials have demonstrated beneficial effects of the addition of bevacizumab to chemotherapy on patients’ clinical conditions. Such combination has significantly reduced the risk of disease progression and death in comparison with chemotherapy only [5–9].

One of the main drawbacks of new oncologic agents is their high cost when compared with conventional chemotherapy. The development, incorporation and use of new technologies in the context of healthcare systems, along with the sustainability of these systems, are inserted in social and economic contexts that reflect the continuous production and consumption of products. This process should be built based on health needs, public budgeting, responsibilities of the three levels of government (including social control), and on the principles of equity, universality and integrity of the Brazilian healthcare system. In light of this, the National Policies in Healthcare Technology Management aims to maximize the benefits obtained from the available resources, so as to guarantee the equal access of the population to effective and safe technologies [10].

The aim of the present study was to perform a cost-effectiveness analysis of bevacizumab plus XELOX and XELOX alone as the first-line therapy of mCRC patients from the perspective of a public hospital school in Brazil.

## Methods

This was a cost-effectiveness analysis performed by decision tree and Markov models. Decision analysis model was constructed using the TreeAge Pro 2013® software [11]. The following chemotherapy regimens were compared:

- XELOX: capecitabine 1000 mg/m^2^ twice a day for 14 days, followed by a week free of treatment; oxaliplatin 130 mg/m^2^ on day 1 [4];
- XELOX plus bevacizumab: capecitabine 1000 mg/m^2^ twice a day for 14 days, followed by a week free of treatment; oxaliplatin 130 mg/m^2^ on day 1; and bevacizumab 7.5 mg/kg on day 1 every three weeks [4].

Costs were expressed in local currency (Brazilian real, BRL), and the outcomes were expressed in months of life gained (MLG).

### Description and structure of the model

Once in treatment, patients were allocated to one of the four states defined by the model: (1) first-line therapy; (2) second-line therapy; (3) supportive care; and (4) death.

The model was based on the following assumptions: 100% of the patients started at the first state (first-line therapy), and stayed at this same state or moved to the others after the first cycle; patients in second-line therapy could stay at this state or moved to supportive care or die; patients in supportive care could stay at this state or die; death was considered the absorption state in the proposed model.

The folinic acid, 5-fluorouracil (5FU) and irinotecan (FOLFIRI) regimen was considered as the second-line therapy in both strategies. The regimen consists in the infusion of irinotecan 180 mg/m^2^ on day 1 for 90 min; a pulse dose of 400 mg/m^2^ of 5FU on day 1 followed by the infusion of 2400 mg/m^2^ for 46 h; and leucovorin 200 mg/m^2^ given as a 2-h infusion every 2 weeks [12].

The perspective included was that of a tertiary, public hospital in Brazil, involved in teaching, research, extension and service, maintained by the Brazilian Unified Health System (SUS) resources, complemented with the Sao Paulo Secretary of Health funds.

The model was constructed in a 60-month time horizon, which allowed the follow-up of all stages of the disease. Each cycle was defined as a three-month period (total of 20 periods). Univariate sensitivity analyses were displayed in a stochastic tornado diagram, showing the variation of the parameters as a function of the distribution of probabilities [13].

### Assessment of costs in health care

Data of health care costs were collected retrospectively using the micro-costing method from the electronic database of the General Hospital of Ribeirao Preto Medical School, University of Sao Paulo between 01 January 2009 and 31 October 2013. The hospital has 875 beds and is qualified, by the Brazilian Ministry of Health, as a healthcare center for highly complex cases in oncology, and is nationally recognized as a center of excellence in teaching, research and services.

We used the method proposed by Drummond et al. [14], in which the real monetary costs of health care are categorized in: medications, laboratory and imaging tests, preparation of chemotherapy drugs by a dedicated pharmacy, and administration of the chemotherapy by the nursing staff.

- *Medications*: the costs of all chemotherapy drugs included in the protocol (including adjuvants) administered to the patients during hospitalization or outpatient care.
- *Laboratory and imaging tests:* the costs of all tests performed during treatment, considering the real costs paid by the hospital, and including consumption materials, equipment and human resources;
- *Preparation of chemotherapy drugs*: the costs of all materials used for the preparation of the infusional therapy per treatment cycle. It also included the mean cost of the manipulation of each infusion bag (except for the drugs), human resources, salary taxes and benefits, and facility-related costs (water, electricity, telephone);
- *Administration of chemotherapy drugs*: the costs of all materials used for the infusion of the chemotherapy drugs, including human resources, salary taxes and benefits, facility-related costs (cleaning and sanitizing costs, dietetics and nutrition, hospital clothes, water, electricity and telephone). For the regimens administered in an outpatient setting (XELOX and XELOX plus bevacizumab), the costs of the patient/day at the chemotherapy unit were included and, for the FOLFIRI protocol, the mean cost of the patient/day during hospitalization at the Clinical Oncology Unit of this hospital were included. These costs were estimated by the chemotherapy staff.

For the FOLFIRI protocol (second-line therapy), a long-term central venous catheter was implanted for the administration of 5-fluorouracil. Only the cost of the catheter (230 BRL for the year of 2013) was included in the analysis, since the costs involved in the implementation of the device were not available.

All costs were estimated from the first until the last day of the chemotherapy protocol plus 30 days thereafter. This 30-day period was included because of possible adverse events from chemotherapy, and to perform final laboratory and imaging tests.

Since data related to drug costs were collected retrospectively, a 5% inflationary adjustment to chemotherapy drugs was made from 2009 to 2013 and presented in BRL. For the other costs, the year of 2013 was used as the reference year, with no inflationary adjustments. To increase the comparability with other studies, a discount rate of 5% a year was adopted, according to the Brazilian Ministry of Health Methodological Guidelines for Economic Analysis [13].

### Characterization and measurement of clinical outcomes

The clinical outcomes assessed were progression-free survival (PFS), defined as the time elapsed between treatment initiation and disease progression or death, in months, and overall survival (OS), and defined as the time elapsed between treatment initiation and death, in months.

A systematic review was performed on PubMed, Cochrane and Lilacs databases, using the terms (“Colorectal Neoplasms”[Mesh]) AND (“XELOX”[Supplementary Concept] OR “bevacizumab”[Supplementary Concept]) AND (“Survival Analysis”[Mesh]) for Pubmed and Cochrane, and the terms “câncer cólon-retal metastático” AND “análise de sobrevida” OR “XELOX” for Lilacs. The search was performed on 09 February 2015.

The inclusion criteria were systematic review or randomized clinical trial on patients with mCRC in palliative treatment, receiving a combination of chemotherapy protocols that included bevacizumab. Exclusion criteria were economic analysis studies, narrative reviews, studies without control groups, pharmacokinetics studies, pharmacodynamics studies, case reports, and case series studies.

Following the reading of the abstracts and analysis for inclusion and exclusion criteria, the selected articles were fully read and analyzed for methodological quality by using the Jadad scale [15] or the AMSTAR [16].

The probability of transition from one state to another was calculated using the PFS and OS estimated by the Kaplan-Meier method in the selected studies.

### Ethical aspects

The study was approved by the local Ethics Committee on April 17th, 2013 (number 956/2013).

## Results

### Quantification and costing of resources

Costs of each health state estimated for a three-month period (1 Markov model cycle) of strategy 1 (XELOX) and strategy 2 (XELOX plus bevacizumab) are described in Tables 1 and 2, respectively.

**Table 1:** Costs estimated for a three-month period (one Markov model cycle) of each health state of the model, in Brazilian real (BRL) and percentage (%) by category in strategy 1 (XELOX)

Category	First-line treatment (XELOX)		Second-line treatment (FOLFIRI)		Supportive care		Death	
	R$	%	R$	%	R$	%	R$	%
Medications	6428.00	77.86	4327.80	33.33	0.00	0.00	0.00	0.00
Preparation (Pharmacy)	81.24	0.98	270.18	2.08	0.00	0.00	0.00	0.00
Administration (Nursing)	580.80	7.03	6700.74	51.61	0.00	0.00	0.00	0.00
Laboratory tests	265.48	3.22	409.08	3.15	265.48	5.28	0.00	0.00
Imaging tests	900.80	10.91	1276.20	9.83	0.00	0.00	0.00	0.00
Hospitalization	0.00	0.00	0.00	0.00	4767.03	94.72	0.00	0.00
Total	8256.32	100.00	12,984.00	100.00	5032.51	100.00	0.00	0.00

*Abbreviations*: *XELOX* Xeloda® and oxaliplatin, *FOLFIRI* 5-fluorouracil, leucovorin and irinotecan

**Table 2:** Costs estimated for a three-month period (one Markov model cycle) of each health state of the model, in Brazilian real (BRL) and percentage (%) by category in strategy 2 (XELOX plus bevacizumab)

Categories	First-line treatment (XELOX plus bevacizumab)		Second-line treatment (FOLFIRI)		Clinical Support		Death	
	R$	%	R$	%	R$	%	R$	%
Medications	27,592.00	89.57	4327.80	33.33	0.00	0.00	0.00	0.00
Preparation (Pharmacy)	120.08	0.39	270.18	2.08	0.00	0.00	0.00	0.00
Administration (Nursing)	1006.40	3.27	6700.74	51.61	0.00	0.00	0.00	0.00
Laboratory tests	367.00	1.19	409.08	3.15	265.48	5.28	0.00	0.00
Imaging tests	1720.00	5.58	1276.20	9.83	0.00	0.00	0.00	0.00
Hospitalization	0.00	0.00	0.00	0.00	4767.03	94.72	0.00	0.00
Total	30,805.48	100.00	12,984.00	100.00	5032.51	100.00	0.00	0.00

*Abbreviations*: *XELOX* Xeloda® and oxaliplatin, *FOLFIRI* 5-fluorouracil, leucovorin and irinotecan

The states “first line-treatment” and “second-line treatment” were analyzed for all cost categories previously defined, and the state “supportive care” was analyzed for “laboratory tests” category. The costs of hospitalization (three days /month) at the Clinical Oncology Unit were also included, considering a value of 529.67BRL/day, estimated based on the reference year 2013.

### Results of the systematic review

A total of 337 citations were found on PubMed, Cochrane and Lilacs databases, 7 were selected for full reading, and three studies [4, 7, 17] were included in our analysis.

To complete the model, data of FOLFIRI protocol were obtained from the study by Tournigand et al. [12], and data from the randomized clinical trial of Van Cutsem et al. [18] were used for extraction of mortality data of patients in supportive care. A summary of these studies are described in Table 3.

**Table 3:** General characteristics of the studies included in the review

Study	Objective	Type of study	No. patients	Outcomes
Macedo et al. (2012) [7]	To collect current data and evaluate the effect of bevacizumab on first-line therapy, focusing on each backbone regimen; subgroup analysis.	Systematic review	3060	PFS and OS
Hurwitz et al. (2013) [17]	To describe the results of the analysis of RCTs on bevacizumab in mCRC. The analysis pooled individual patient data from these studies, which allowed a more comprehensive examination of efficacy and safety of bevacizumab.	Systematic review	3763	PFS and OS
Saltz et al. (2008) [4]	To evaluate the efficacy and safety of bevacizumab when added to oxaliplatin in first-line therapy (capecitabin plus oxaliplatin [XELOX] or fluorouracil/folinic acid plus oxaliplatin [FOLFOX]) in mCRC patients.	RCT	1401	PFS and OS
Tournigand et al. (2004) [12]	To evaluate FOLFIRI and FOLFOX regimens to determine the best sequence (FOLFIRI or FOLFOX first) to treat mCRC patients.	RCT	220	PFS and OS
Van Cutsem et al. (2007) [18]	To compare panitumumab plus supportive care versus supportive care in mCRC patients who had progressed after standard chemotherapy.	RCT	463	PFS and OS

*Abbreviations*: *XELOX* Xeloda® and oxaliplatin, *FOLFIRI* 5-fluorouracil, leucovorin and irinotecan, *FOLFOX* 5-fluorouracil, leucovorin and oxaliplatin, *RCT* randomized, clinical trial, *mCRC* metastatic colorectal cancer, *PFS* progression free survival, *OS* overall survival

The three studies included in this review were of high methodological quality according to the AMSTAR method [16] and the Jadad scale [15], with a score between 3 and 5.

Parameters of effectiveness, costs and transition probabilities, as well as the variations in sensitivity analysis are described in Table 4. The variation ranges were established based on the analyzed studies or on the assumption of a 10% variation. For ‘costs’, the variation range was determined by its standard deviation.

**Table 4:** Effectiveness, costs, transition probabilities, base case discount rate and variations in sensitivity analysis

Parameters	Base case	Variation in sensitivity analysis	Reference
Overall survival of first-line therapy (strategy 1)	17.6	15.8–19.3	Macedo et al. (2012) [7]
Overall survival of second-line therapy (strategy 1)	20.6	18.5–22.6	Tournigand et al. (2004) [12]
Overall survival of supportive care (strategy 1)	18	16.2–19.8	Van Cutsem et al. (2007) [18]
Overall survival of first-line therapy (strategy 2)	19.8	17.8–21.8	Macedo et al. (2012) [7]
Overall survival of second-line therapy (strategy 2)	20.6	18.5–22.6	Tournigand et al. (2004) [12]
Overall survival of supportive care (strategy 2)	18	16.2–19.8	Van Cutsem et al. (2007) [18]
Cost of first-line therapy (strategy 1)	8256.32	6636.06–9876.58	Assumed
Cost of second-line therapy (strategy 1)	12,984.00	11,600.7–14,367.3	Assumed
Cost of supportive care (strategy1)	5032.51	4529.26–5535.76	Assumed
Cost of first-line therapy (strategy 2)	30,805.48	20,664–40,946.96	Assumed
Cost of second-line therapy (strategy 2)	12,984.00	11,600.7–14,367.3	Assumed
Cost of supportive care (strategy2)	5032.51	4529.26–5535.76	Assumed
Probability of transition from first-line therapy to first-line therapy (strategy 1)	0.66	0.60–0.72	Assumed
Probability of transition from first-line therapy to second-line therapy (strategy 1)	0.2	0.18–0.22	Hurwitz et al. (2013) [17]
Probability of transition from first-line therapy to supportive care (strategy 1)	0.08	0.07–0.09	Saltz et al. (2008) [4]
Probability of transition from first-line therapy to death (strategy 1)	0.06	0.04–0.08	Hurwitz et al. (2013) [17]
Probability of transition from second-line therapy to second-line therapy (strategy 1)	0.49	0.45–0.53	Tournigand et al. (2004) [12]
Probability of transition from second-line therapy to supportive care (strategy 1)	0.45	0.41–0.49	Tournigand et al. (2004) [12]
Probability of transition from second-line therapy to death (strategy 1)	0.06	0.04–0.08	Tournigand et al. (2004) [12]
Probability of transition from supportive care to supportive care (strategy 1)	0.75	0.68–0.82	Van Cutsem et al. (2007) [18]
Probability of transition from supportive care to death (strategy 1)	0.25	0.23–0.27	Van Cutsem et al. (2007) [18]
Probability of transition from first-line therapy to first-line therapy (strategy 1)	0.67	0.61–0.73	Assumido
Probability of transition from first-line therapy to second-line therapy (strategy 1)	0.12	0.10–0.14	Hurwitz et al. (2013) [17]
Probability of transition from first-line therapy to supportive care (strategy 1)	0.16	0.14–0.18	Saltz et al. (2008) [4]
Probability of transition from first-line therapy to death (strategy 1)	0.05	0.04–0.06	Hurwitz et al. (2013) [17]
Probability of transition from second-line therapy to second-line therapy (strategy 1)	0.49	0.45–0.53	Tournigand et al. (2004) [12]
Probability of transition from second-line therapy to supportive care (strategy 1)	0.45	0.41–0.49	Tournigand et al. (2004) [12]
Probability of transition from second-line therapy to death (strategy 1)	0.06	0.04–0.08	Tournigand et al. (2004) [12]
Probability of transition from supportive care to supportive care (strategy 1)	0.75	0.68–0.82	Van Cutsem et al. (2007) [18]
Probability of transition from supportive care to death (strategy 1)	0.25	0.23–0.27	Van Cutsem et al. (2007) [18]
Discount rate	5%	0–10%	Brasil, 2014 [13]

### Cost-effectiveness analysis

The analysis of the model proposed resulted in an incremental difference of 2.25 MLG for a cost of 47,833.57BRL, with an incremental cost-effectiveness ratio (ICER) of 21,231.43 per MLG. Table 5 shows the estimated ICER for the cohort.

**Table 5:** Results of cost-effectiveness analysis (BRL/ months of life gained)

Strategy	Cost (R$)	Incremental cost (R$)	Effectiveness (MLG)	Incremental effectiveness (MLG)	ICER (BRL/MLG)
XELOX	41,396.84		97.07		
XELOX + bevacizumab	89,230.41	47,833.57	99.32	2.25	21,231.43

*Abbreviations*: *ICER* Incremental cost-effectiveness ratio, *MLG* months of life gained, *XELOX* Xeloda® and oxaliplatin; *BRL* Brazilian real

### Sensitivity analysis

**Fig. 1:**
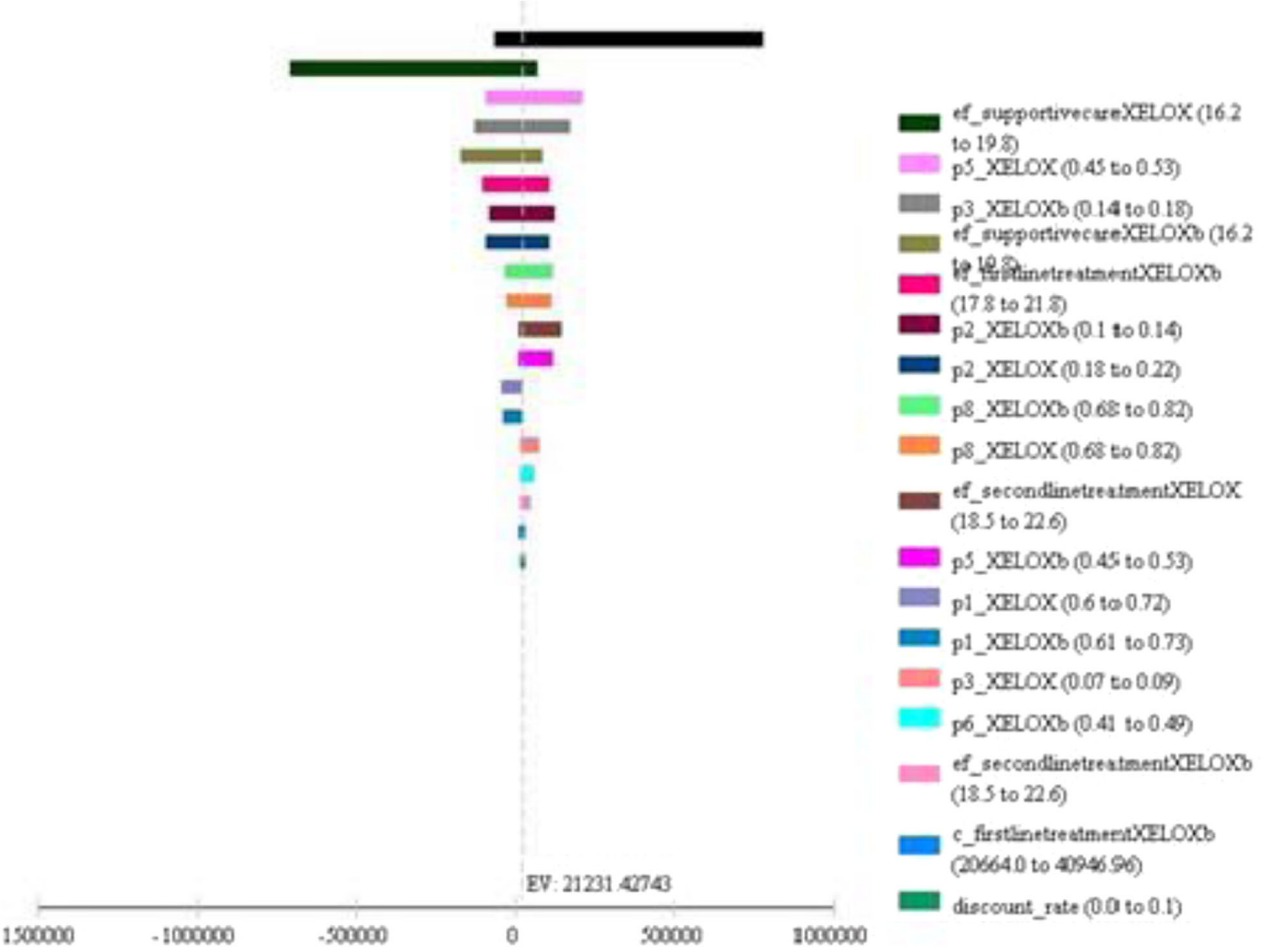
Tornado diagram showing incremental cost-effectiveness ratio after the inclusion of minimum and maximum values of the parameters to the model

The tornado diagram graphically and simultaneously displays the sensitivity of many parameters (Fig. 1). In strategy 1, “effectiveness” had the greatest impact on “supportive care” state. The ICER varied from 7814.47BRL to 29,614.12BRL for the minimum and maximum effectiveness value, respectively, indicating that strategy 2 was dominated by the strategy 1.

**Fig. 2:**
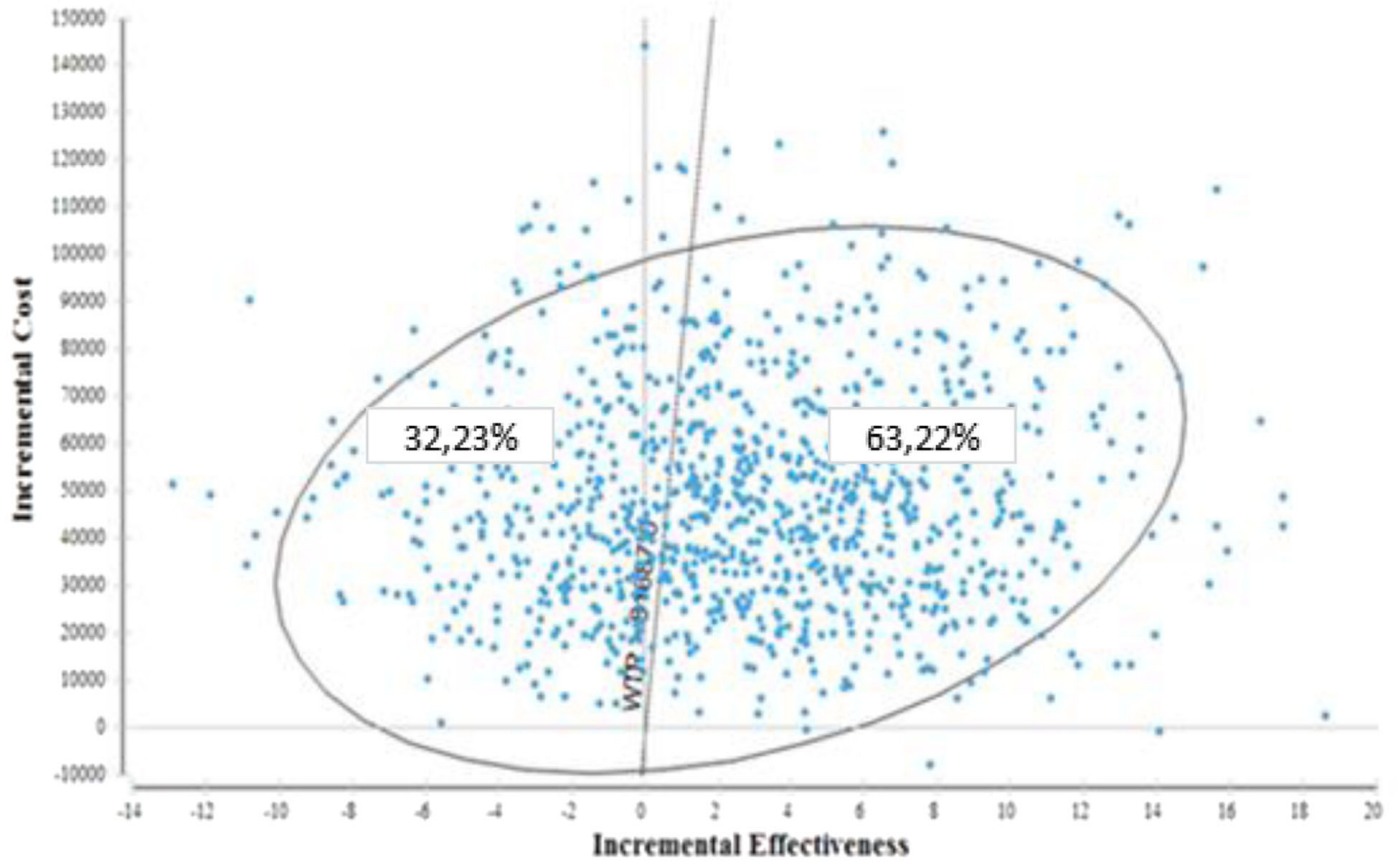
Results of probabilistic sensitivity analysis

Probabilistic sensitivity analysis was performed using the Monte Carlo simulation, in which variables change according to pre-established probability distributions (Fig. 2). A gamma distribution and a uniform distribution were used for the parameters of cost and effectiveness, respectively. A total of 100,000 simulations were performed and, in each simulation, a set of values for each parameter was randomly drawn from the distribution. Using the WHO criteria [14], the gross domestic product (GDP) per capita in Brazil was 27,229BRL in 2014, with a maximum willingness to pay threshold of 81,687BRL for three GDP per capita.

Figure 2 shows that 63.22% of the results are in quadrant 1, with a positive, increasing incremental effectiveness and cost, and 32.23% are in quadrant 2, with a negative, decreasing incremental effectiveness. The other results are in quadrant 3 and 4.

**Fig. 3:**
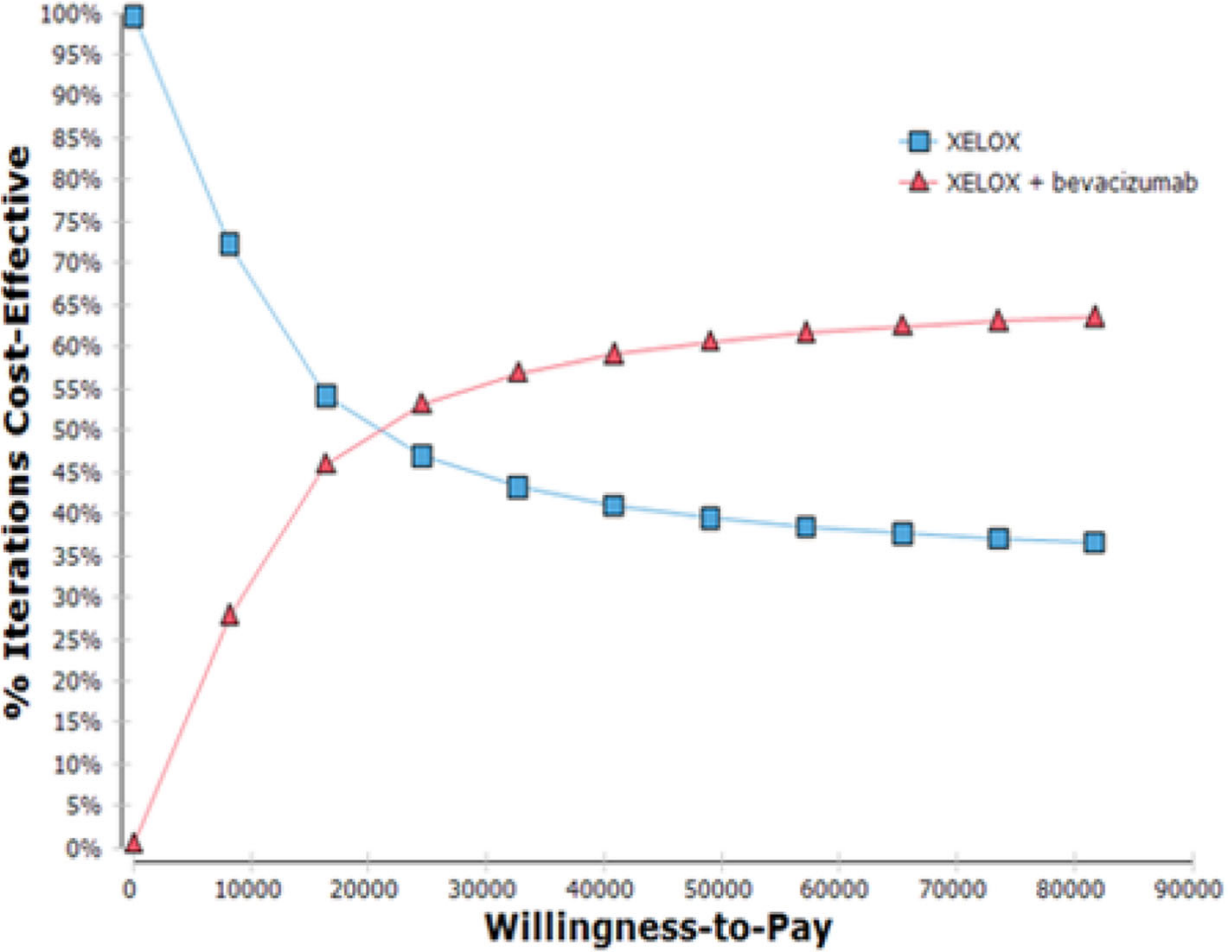
Acceptability curve of the Markov model comparing the strategies XELOX and XELOX plus bevacizumab used in the first line in the treatment of patients with metastatic colorectal cancer

The uncertainty about cost-effectiveness results is also presented in Fig. 3. We can show that from a willingness to pay threshold of 21,231.43BRL per MVG, the probability that strategy 2 (XELOX plus bevacizumab) will be more cost-effective than strategy 1. When this threshold reaches its maximum value of 81,687BRL, according to WHO (three GDP per capita), this probability is 63.5% for strategy 2 and 36, 5% for strategy 1 for the year 2014.

## Discussion

Costs of oncology drugs have caused a considerable impact on Brazilian public budgeting, particularly due to development of biotechnology, which has sparked a revolution in cancer treatment. This has caused a drastic increase in treatment costs, without necessarily indicating the feasibility of public health system in incorporating these medications.

In Brazil, the National Commission for the Incorporation of Technologies (*Comissão Nacional de Incorporação de Tecnologias no SUS*, CONITEC), created by the law 12,401 on April 28th 2011, addresses therapeutic assistance and incorporation of health technology in the scope of the SUS. Its aim is to advise the Ministry of Health in the incorporation, exclusion or changes in health technologies, as well as in the development or updates of clinical protocols and therapeutic guidelines based on economic analysis studies [19].

Considering the available literature, this is the first study aimed to conduct an economic analysis comparing the costs of XELOX and XELOX plus bevacizumab from the perspective of the public health system.

This study has some limitations that should be considered. This model did not examine the possibility of patients move from a second-line therapy to a third-line therapy due to advanced stages of the disease. Because of diagnostic delay and limited access to an oncology center, patients may start treatment late. For this reason, we decided to include the state “supportive care”, as many patients cannot continue treatment or start a new line of treatment due to clinical conditions.

Tappenden et al. [20] estimated the cost-effectiveness of adding bevacizumab to 5FU, irinotecan and leucovorin in comparison with 5FU and leucovorin alone in patients with mCRC. The states of the model used by the authors were: (1) alive without disease progression; (2) alive with disease progression; and (3) death. Goldstein et al. [21] developed two Markov models to compare costs and effectiveness of bevacizumab in first-line and second-line therapies in the USA. In the first-line therapy, the authors compared FOLFOX with and without bevacizumab in patients recently diagnosed with mCRC and in disease progression. Both groups received FOLFIRI without bevacizumab and progressed to death. In the second-line therapy, FOLFIRI with and without bevacizumab were compared with subsequent progression to death, in patients who had experienced progression during first-line therapy with bevacizumab. Thus, the states were first-line therapy, second-line therapy and death.

The recent study of Franken et al. [22] evaluated the cost-effectiveness of capecitabine and bevacizumab (CAP-B) maintenance compared with the observational strategy following first-line capecitabine, oxaliplatin and bevacizumab (CAPOX-B) induction treatment for mCRC patients with stable disease or better after 6 cycles of treatment. CAP-B maintenance compared with observation resulted in an ICER of €175,452 per quality-adjusted life years (QALY) and €204,694 per life year (LY). Varying the difference in health-related quality of life between CAP-B maintenance and observation influenced the ICER most. For patients achieving complete or partial response on capecitabine, oxaliplatin and bevacizumab induction treatment, an ICER of €149,300 per QALY was calculated.

In Brazil, Carvalho et al. [23] evaluated cost-effectiveness of two treatment strategies in mCRC from the perspective of SUS before and after revision of the values covered by the system, available at the Authorization for Highly Complex Procedures (AHCP) table. The pre-review strategy included 5FU and leucovorin (first-line therapy) followed by irinotecan (second-line therapy). The post-review (with coverage values updated) strategy included FOLFOX (first-line therapy) followed by FOLFIRI (second-line therapy). After the second-line therapy, patients could experience a progress to supportive care and subsequent death, which is similar to our study.

In the present study, the costs of the strategies were estimated using the micro-costing method, aiming to obtain precise information of the real costs paid by the patients in a tertiary, public hospital that offers highly complex care. These estimates may be subject to variations, since the values included in the analysis, registered in the electronic database of this hospital in 2013, were resultant from public bidding. However, these variations were included in the sensitivity analysis.

Our findings showed that in XELOX and XELOX plus bevacizumab regimens, the greatest impact on total treatment cost was caused by medications. It is worth mentioning oral capecitabine, which is an available, effective, safe treatment option for mCRC, requires lower number of chemotherapy sessions and promotes better adaptation to the treatment proposed [24].

Due to reduced number of hospital beds, hospitalization for chemotherapy is often unavailable for SUS beneficiaries. Besides, there are not infusion pumps for these patients to receive chemotherapy at home. These factors contribute for delays in the treatment proposed [25].

In addition, the cost analysis revealed that the category that had the greatest impact on FOLFIRI regimen was the cost of administration (51.6%) rather than the cost of medications (33.3%). The use of infusion pumps would hence be an alternative strategy to reduce these costs and adjust them to the values covered by the SUS (AHCP table). Tampellini [26] compared the costs of the administration of FOLFIRI and FOLFOX regimens in ambulatorial setting using an infusion pump with the administration in the hospital setting. In a same time period and with the same resources, the infusion pump permitted treatment of at least five times more patients than the traditional treatment at the hospital.

In the study by Carvalho et al. [23], costs related to drugs, laboratory and radiology tests, medical fees, and hospitalization were obtained from the official prices regulated by the Ministry of Health. Other parameters, including the number of visits were obtained from an opinion survey of oncologists of public health centers. The cost estimated by the authors for a three-month treatment with FOLFIRI was 13,925 BRL, which was similar to that found in our study (12,984 BRL). We also included the costs of medications used for possible adverse effects from the treatment.

In an economic analysis conducted with head and neck squamous cell carcinoma patients, Brentani [27] used the SUS coverage values and pointed out the difficulty in obtaining data of costs, as well as the absence of indirect cost data in patients’ medical records.

According to the AHCP table, the reimbursement value of first-line palliative chemotherapy of colon and rectal adenocarcinoma (locoregionally advanced, metastatic or recurrent disease) was 2224 BRL per month. Today, 80% of services provided to cancer patients in Brazil were performed by the public health system [28]. Therapy drugs are not specified in the AHCP table, and treatment choice is a medical staff’s decision, based on local protocols and international scientific guidelines.

Due to the lack of Brazilian studies on this subject, data of treatment effectiveness were obtained from international studies. Also, data of quality of life were unavailable in most of these studies, and hence only PFS and OS data were used in the analysis, which make it difficult to compare our results with those of studies that used the QALY outcome.

The decision to incorporate a new technology into the public health system depends on how much the beneficiaries would be willing to pay for the additional benefit. We found an ICER of 21,231.43BRL per MLG, which would correspond to 254,777.16 BRL per life year gained. In the study by Carvalho et al. [23], the cost of the implementation of FOLFOX and FOLFIRI regimens was 78,188 BRL per life year gain and, hence, not cost-effective when compared with 5FU plus leucovorin. Nevertheless, the authors brought up for discussion the fact that the current Brazilian health system reimbursement model does not permit the incorporation of new regimen protocols in a cost-effective manner.

The ICER of bevacizumab could be improved by the use of an effective biomarker to identify those patients who are more likely to benefit from treatment. For example, KRAS mutation testing identifies which patients with mCRC would benefit more from treatments such as cetuximab or panitumumab, increasing the cost-effectiveness of these interventions [29].

Our findings make several contributions: first, the study presents real health care costs related to mCRC treatment in a large, public hospital that offers highly complex care, which could be used for the planning and elaboration of public health policies. Second, it brings up for discussion the necessity of the AHCP table revision to permit the incorporation of cost-effective biological medications in the SUS. Finally, the results of this economic analysis, performed by a modeling method, provide valuable information on how financial resources can be efficiently allocated in the analysis of new strategies for the treatment of the Brazilian public health system beneficiaries.

## Conclusion

The cost-effective analysis of XELOX (strategy 1) and XELOX plus bevacizumab (strategy 2) as first-line treatment of mCRC patients from the perspective of a public hospital resulted in a ICER of 21,231.43 BRL per MLG, or 254,777.16 BRL per life year gained. Therefore, the SUS reimbursement values do not allow the inclusion of bevacizumab to the treatment of mCRC patients in a cost-effective manner.

## Abbreviations

AHCP: Authorization for highly complex procedures
AMSTAR: Assessment of multiple systematic reviews
BRL: Brazilian real
CAP-B: Capecitabine and bevacizumab
CAPOX-B: Capecitabine, oxaliplatin and bevacizumab
CONITEC: *Comissão Nacional de Incorporação de Tecnologias no SUS*
CRC: Colorectal cancer
FOLFOX: Folinic acid, 5-fluorouracil and oxaliplatin
Folinic acid: 5-fluorouracil and irinotecan
GDP: Gross domestic product
ICER: Incremental cost-effectiveness ratio
LY: Life year
mCRC: Metastatic colorectal cancer
MLG: Months of life gained
OS: Overall survival
PFS: Progression-free survival
QALY: Quality adjusted life year
SUS: Brazilian Unified Health System
VEGF: Vascular endothelial growth factor
XELOX: Xeloda^®^ and oxaliplatin; 5-FU: 5-fluorouracil
